# Infant Saliva Levels of microRNA miR-151a-3p Are Associated with Risk for Neurodevelopmental Delay

**DOI:** 10.3390/ijms24021476

**Published:** 2023-01-12

**Authors:** Steven D. Hicks, Alexandra Confair

**Affiliations:** Penn State College of Medicine, Department of Pediatrics, 500 University Drive, Hershey, PA 17033, USA

**Keywords:** non-coding RNA, epigenetic, pediatric, development, prognostic, miRNA, child, screening, non-invasive, biomarker

## Abstract

Prompt recognition of neurodevelopmental delay is critical for optimizing developmental trajectories. Currently, this is achieved with caregiver questionnaires whose sensitivity and specificity can be limited by socioeconomic and cultural factors. This prospective study of 121 term infants tested the hypothesis that microRNA measurement could aid early recognition of infants at risk for neurodevelopmental delay. Levels of four salivary microRNAs implicated in childhood autism (miR-125a-5p, miR-148a-5p, miR-151a-3p, miR-28-3p) were measured at 6 months of age, and compared between infants who displayed risk for neurodevelopmental delay at 18 months (*n* = 20) and peers with typical development (*n* = 101), based on clinical evaluation aided by the Survey of Wellbeing in Young Children (SWYC). Accuracy of microRNAs for predicting neurodevelopmental concerns at 18 months was compared to the clinical standard (9-month SWYC). Infants with neurodevelopmental concerns at 18 months displayed higher levels of miR-125a-5p (d = 0.30, *p* = 0.018, adj *p* = 0.049), miR-151a-3p (d = 0.30, *p* = 0.017, adj *p* = 0.048), and miR-28-3p (d = 0.31, *p* = 0.014, adj *p* = 0.048). Levels of miR-151a-3p were associated with an 18-month SWYC score (R = −0.19, *p* = 0.021) and probability of neurodevelopmental delay at 18 months (OR = 1.91, 95% CI, 1.14–3.19). Salivary levels of miR-151a-3p enhanced predictive accuracy for future neurodevelopmental delay (*p* = 0.010, X^2^ = 6.71, AUC = 0.71) compared to the 9-month SWYC score alone (OR = 0.56, 95% CI, 0.20–1.58, AUC = 0.567). This pilot study provides evidence that miR-151a-3p may aid the identification of infants at risk for neurodevelopmental delay. External validation of these findings is necessary.

## 1. Introduction

Neurodevelopmental delay can be defined as the acquisition of speech, gross motor, fine motor, or social milestones that deviates from typical patterns of infancy or childhood development [1]. Early detection of neurodevelopmental delay is a cornerstone of pediatric medical care [2], because prompt initiation of intervention services during periods of brain plasticity has been shown to buttress developmental trajectories [3]. Neurodevelopmental surveillance is typically performed at regularly scheduled check-ups, using standardized screening surveys that rely on caregiver reports [4]. However, this approach has several limitations that can hinder the accurate recognition of neurodevelopmental delays.

Written neurodevelopmental surveys require a basic level of parent/guardian literacy [5]. Further, the accuracy of milestone reporting relies on the familiarity of caregivers with child behaviors [6]. This accuracy may be impacted by socioeconomic and cultural factors [7,8,9,10]. For example, a surveillance checklist that asks about a specific song or play activity may not be recognized by all caregivers. Neurodevelopmental screening tools that lack sensitivity within certain cultures may bias referral and treatment decisions for minority groups [11,12]. Conversely, tools that lack specificity may delay diagnoses by exacerbating wait times for specialist evaluation [13].

Biomarkers of neurodevelopmental delay represent a novel, objective way to aid developmental assessment. One emerging category of neurodevelopmental biomarkers includes non-coding ribonucleic acids (RNAs), such as micro-ribonucleic acids (miRNAs) [14]. These small molecules have the ability to link genetic and environmental contributions to neurodevelopment by regulating the translation of messenger RNA into functional proteins. In vitro and in vivo studies have established the importance of miRNA activity in brain development [15,16]. A growing body of literature has also demonstrated that miRNA levels in the human body are associated with cognition, learning, and neurodevelopmental disorders [17,18,19,20]. The ability of neurons to extrude miRNAs within protective extracellular vesicles as a means of intracellular communication renders these molecules ideal biomarkers of neurodevelopmental delay [14,21].

Saliva is an abundant source of exosome miRNAs [22,23]. Salivary exosomes arise, in part, from neurons, such as those populating cranial nerves within the oropharynx [24,25]. Salivary miRNA levels have been shown to correlate with measures of adaptive behaviors in children [17,26]. In young children with autism, some salivary miRNA perturbations persist into adolescence, while other miRNA levels moderate alongside gains in adaptive behavior [18]. Previously we demonstrated that levels of four saliva miRNAs could be used to differentiate 224 children with autism spectrum disorder from 219 peers with typical development or neurodevelopmental delay (ages 2–6 years) [27]. It remains unclear whether perturbations in these miRNAs differentiate children with neurodevelopmental delay from peers with typical development, or if they precede clinical signs of neurodevelopmental delay in infancy. If so, saliva miRNAs could serve as non-invasive biomarkers for neurodevelopmental surveillance.

The objective of this study was to assess whether saliva miRNA levels could be used to identify infants at risk for future neurodevelopmental delays. We hypothesized that the four saliva miRNAs previously identified as biomarker candidates in preschool children with autism would display prognostic utility for future neurodevelopmental delay in infancy [27]. This hypothesis was tested in a prospective cohort study of 121 term infants.

## 2. Results

### 2.1. Participants

Participating infants were predominantly female (71/121, 59%), white race (91/121, 75%), and non-Hispanic (102/121, 84%), with an average gestational age of 39 weeks and an average birth weight of 3370 (±425) grams (Table 1). Relatively few infants received public health insurance (17/121, 14%), or lived in a single parent household (21/121, 17%), a home built prior to 1977 (28/121, 24%), or a home with household income <USD 25,000 (5/121, 5%). The average household size was four persons (range: 2–9). There were no significant differences in medical/demographic characteristics or social determinants of health between infants who displayed typical development at 18 months (*n* = 101) and peers who displayed risk factors for neurodevelopmental delay (*n* = 20).

Among all participants, the mean neurodevelopmental score on the SWYC at 18 months of age was 13 (±4; range: 5–20). Those at risk for neurodevelopmental delay displayed significantly lower mean scores (7 ± 1) compared to peers with typical development (14 ± 3; *p* = 1.7 × 10^−^^16^). The majority of participants who clinicians identified as at-risk for neurodevelopmental delay (19/20, 95%) displayed a “needs review” score on the SWYC. The proportion of participants at risk for neurodevelopmental delay (20/121, 16.5%) was consistent with published rates of neurodevelopmental delay [2,4,5,6,7].

### 2.2. Saliva miRNA Levels

**Figure 1:**
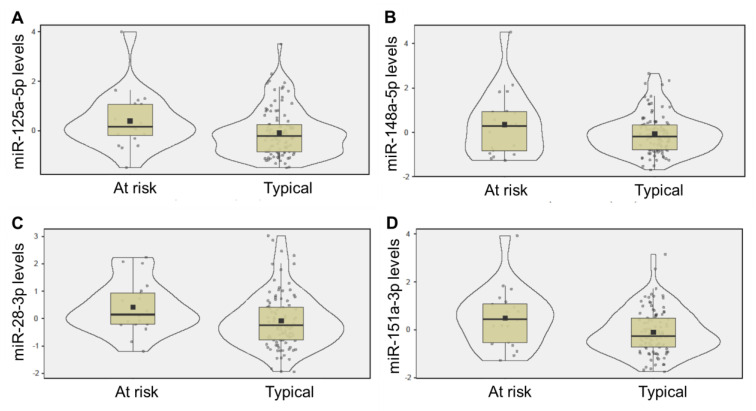
Saliva miRNAs levels display neurodevelopment-specific patterns in infants prior to clinical presentation of developmental concerns. The violin box plots display salivary levels of miR-125a-5p (**A**), miR-148a-5p (**B**), miR-28-3p (**C**), and miR-151a-3p (**D**), at 6 months of age. Levels of miR-125a-5p (*p* = 0.018), miR-28-3p (*p* = 0.014), and miR-151a-3p (*p* = 0.017) were elevated in the group of infants that went on to display at-risk neurodevelopmental patterns at 18 months of age (*n* = 20), compared to peers with typical development (*n* = 101). Mean (black square), median (black line), and standard deviation (box) are displayed.

**Figure 2:**
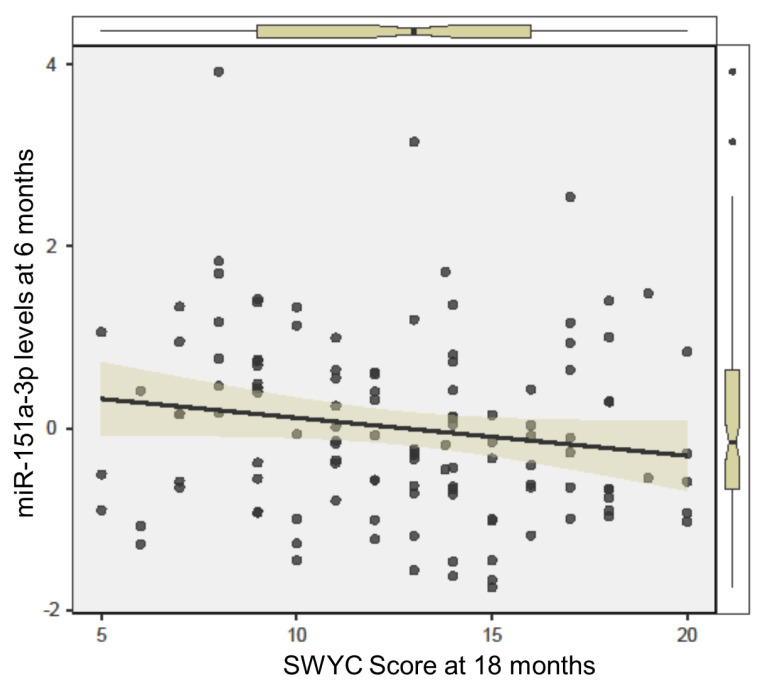
Salivary levels of miR-151a-3p at 6 months are associated with neurodevelopmental score at 18 months. The scatterplot displays sum-normalized levels of miR-151a-3p in infant saliva (measured at 6 months), relative to standardized neurodevelopmental score at 18 months (as measured by the Survey of Wellbeing in Young Children; SWYC). Spearman rank correlation testing displayed a significant association (R = −0.19, *p* = 0.021) between miR-151a-3p levels and SWYC scores.

The four miRNA candidates (miR-125a-5p, miR-148a-5p, miR-151a-3p, miR-28-3p) were each present in 100% of saliva samples, with average raw read counts of 217 (±113), 6923 (±170), 983 (±918), and 24,787 (±268), respectively. Levels of miR-125a-5p (d = 0.30, *p* = 0.018, adj *p* = 0.049), miR-151a-3p (d = 0.30, *p* = 0.017, adj *p* = 0.048), and miR-28-3p (d = 0.31, *p* = 0.014, adj *p* = 0.048) displayed significant differences between infants who later developed at-risk neurodevelopmental patterns and peers who displayed typical development. At six months of age, all three miRNAs were higher in the saliva of infants at risk for neurodevelopmental delay (Figure 1). Salivary levels of miR-151a-3p at 6 months of age were inversely associated with neurodevelopmental score at 18 months of age (R = −0.19, *p* = 0.021) (Figure 2).

### 2.3. Predicting Neurodevelopmental Delay

**Figure 3:**
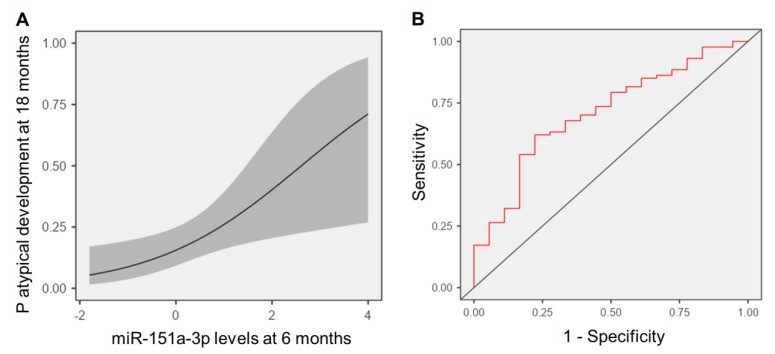
Salivary levels of miR-151a-3p at 6 months aid prediction of neurodevelopmental delay at 18 months. The marginal means plot (**A**) displays the relationship between saliva levels of miR-151a-3p and the probability (P) of atypical development at 18 months (OR = 1.91, 95% CI = 1.14–3.19). The receiver operating characteristic curve (**B**) shows the accuracy of a logistic model coupling salivary levels of miR-151a-3p at 6 months with developmental score on the Survey of Wellbeing in Young Children at 9 months, for predicting future neurodevelopmental delay at 18 months. The algorithm displayed 83% sensitivity and 51% specificity, with an area under the curve of 0.71.

The ability of saliva miRNA levels at six months to identify infants at risk for future neurodevelopmental delay was compared against the current clinical standard—assessment with a standardized screening tool (i.e., SWYC score at nine months of age). A logistic regression employing 9-month SWYC scores for each infant was not highly predictive of neurodevelopmental delay concerns at 18 months (*p* = 0.28, X^2^ = 1.17, OR = 0.56, 95% CI = 0.20–1.58, AUC = 0.567). Among the four miRNA candidates, only miR-151a-3p displayed a significant association with future risk for neurodevelopmental delay (*p* = 0.020, X^2^ = 5.43, OR = 1.91, 95% CI = 1.14–3.19). The addition of miR-151a-3p levels to SWYC scores significantly improved prognostic ability (*p* = 0.010, X^2^ = 6.71, AUC = 0.71), yielding 83% sensitivity and 51% specificity (Figure 3). Notably, a model employing social determinants of health (infant race, maternal education, single parent household, health insurance, home age, household income, household size) displayed modest utility for identifying infants at risk for neurodevelopmental delay at 18 months (*p* = 0.085, X^2^ = 13.9, AUC = 0.72). Household income < USD 25,000 (*p* = 0.029, X^2^ = 4.78) and public health insurance (*p* = 0.013, X^2^ = 6.19) were the only factors associated with increased likelihood of neurodevelopmental concern at 18 months, but neither factor modulated miR-151a-3p levels (income *p* = 0.079, X^2^ = 5.09; insurance *p* = 1.0, X^2^ = 2.31 × 10^−7^), or aided predictive accuracy of the SWYC/miR-151a-3p model (income *p* = 0.32, X^2^ = 0.96; insurance *p* = 0.079, X^2^ = 3.08).

### 2.4. Assessing Longitudinal Changes in miR-151a-3p

To explore whether miR-151a-3p represents a “state” or “trait” biomarker, one-month and six-month saliva samples were compared within a subset of infants for whom repeated samples were available (*n* = 47). This information is crucial for understanding whether miR-151a-3p might be predictive of neurodevelopmental delay at even earlier ages, and for determining if miR-151a-3p levels can be leveraged to assess the efficacy of therapeutic behavioral interventions. A within-subjects analysis of variance (ANOVA) displayed no significant changes in saliva miR-151a-3p levels between one and six months of age (*p* = 0.66, X^2^ = 0.19), suggestive of a trait biomarker.

## 3. Discussion

This study of 121 term infants identified three miRNAs (miR-125a-5p, miR-151a-3p, miR-28-3p) with perturbations in salivary levels at 6 months that preceded concerns of neurodevelopmental delay at 18 months. One of the miRNAs (miR-151a-3p) was associated with neurodevelopmental score on the SWYC at 18 months, and aided the prediction of neurodevelopmental concerns. High levels of miR-151a-3p at 6 months were associated with a nearly two-fold increase in the probability of neurodevelopmental concerns at 18 months, exceeding the prognostic utility of standardized screening with the SWYC at 9 months.

The current standard of care for neurodevelopmental surveillance involves routine screening with validated tools, such as the SWYC [28]. The American Academy of Pediatrics recommends that such tools be used at 9, 18, and 30 months of age [29]. However, some pediatricians fail to effectively implement this approach, and children who go on to display atypical neurodevelopment can still be missed at the earliest ages [30]. Indeed, the observation in the current study that neurodevelopmental score at 9 months does not accurately predict risk of neurodevelopmental concerns at 18 months is consistent with limitations noted in some previous cohort studies [31,32,33].

Social determinants of health, such as economic stability, healthcare access, social community connections, neighborhood/built environment, and educational access may all impact child neurodevelopment [34,35,36]. In this cohort, public health insurance and household income <USD 25,000 increased the likelihood of neurodevelopmental concern at 18 months. However, neither factor interacted with miR-151a-3p levels or aided prognostic accuracy of miR-151a-3p. This result is consistent with the observation that miR-151a-3p levels remained stable from 1 month to 6 months of age, suggesting that miR-151a-3p may serve as a static marker of neurodevelopmental traits, rather than a dynamic biomarker that responds to environmental insults, or behavioral therapies [17].

Although miR-151a-3p is expressed in tissues throughout the human body, it plays an important role in cell-cycle and inflammatory pathways that can impact brain development and neuronal function [37,38,39,40]. For example, microglia-derived vesicular miR-151a-3p has been shown to attenuate neuron apoptosis by regulating the p53 pathway [41]. Previous studies examining children with autism have identified perturbations in the levels of miR-151a-3p in serum [42,43], as well as saliva [27]. The directionality of miR-151a-3p differences varies across studies, perhaps as a function of child age, body fluid type, or autism phenotype. In this study, the observation that salivary miR-151a-3p levels are elevated in infants at risk for future neurodevelopmental delay dovetails with prior observations of miR-151a-3p as a repressor of neuronal apoptosis [44], and evidence that children with certain neurodevelopmental conditions have increased synaptic connections, or macrocephaly [44,45]. It remains unclear whether measurement of miR-151a-3p at earlier ages would have additional prognostic utility.

A number of modalities have been proposed for surveying neurodevelopmental risk, including neuroimaging, electroencephalogram, and blood-based biomarkers [46,47,48]. This pilot study suggests that salivary miRNA levels may also represent an adjunctive tool for neurodevelopmental surveillance. Potential advantages of saliva miRNA include non-invasive sample collection that can be completed at home, and reduced healthcare costs [49]. Families of children with autism and neurodevelopmental delays prefer saliva as a biofluid for genetic/epigenetic testing [50], and the ubiquity of saliva-based polymerase chain reaction tests arising from the SARS-CoV-2 pandemic has accelerated the clinical adoption of this technology in medical settings [51].

This study has several limitations. The sample size is small, and requires validation with a larger external cohort. The current cohort involves term infants from predominantly white families, enrolled at a single academic medical center. The results may not generalize to racially or geographically diverse populations, or infants with elevated neurodevelopmental risk factors (e.g., premature infants). The tool that was used to aid detection of neurodevelopmental delay (i.e., the SWYC) is standardized, but does not differentiate fine motor, gross motor, communication, and cognitive delays. Therefore, no assumptions can be made about the relevance of miR-151a-3p to particular neurodevelopmental conditions.

In conclusion, this study examined four miRNAs previously implicated in detection of childhood autism spectrum disorder, and identified one salivary miRNA (miR-151a-3p) whose levels at 6 months of age were directly associated with probability of neurodevelopmental concerns at 18 months. Measurement of salivary miR-151a-3p enhanced prediction of neurodevelopmental risk when added to the clinical standard of care (i.e., neurodevelopmental screening with the SWYC at 9 months). Validation of this approach in a larger, more diverse population could lead to a novel, non-invasive measure aiding the early detection of neurodevelopmental delay and providing opportunities for earlier therapeutic intervention.

## 4. Materials and Methods

### 4.1. Study Design

A convenience sample of 221 infants were enrolled in this prospective longitudinal cohort study. Eligibility criteria included term delivery (37–42 weeks gestation) and plans to breastfeed beyond four months. Exclusion criteria were maternal conditions that could impact breastfeeding ability (e.g., drug addiction, HIV), infant conditions that could impact breastfeeding ability (e.g., cleft lip/palate, metabolic disease, prolonged NICU admission >7 days), factors affecting long-term follow up (e.g., plan for primary pediatric care outside our medical center, plan for infant adoption), and inability to complete standardized surveys (i.e., non-English speaking).

### 4.2. Enrollment

**Figure 4:**
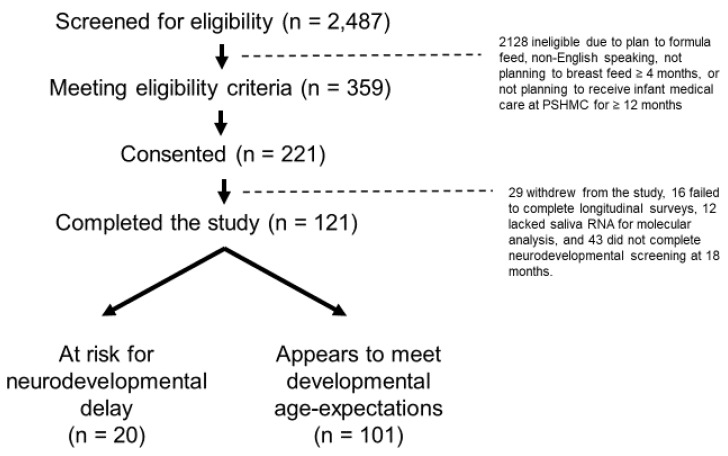
There were 2487 mother–infant dyads screened, 359 eligible dyads approached, and 221 dyads consented. There were 121 infants for whom 6-month saliva samples were available and neurodevelopmental status could be confirmed at 18 months. Pediatricians used the Survey of Wellbeing in Young Children (SWYC) to aid identification of 20 infants as “at risk for neurodevelopmental delay”, and identify 101 infants who “appeared to meet developmental age expectations”. SWYC scores and pediatrician assessments were extracted from the electronic medical record by research staff.

Enrollment occurred between 20 April 2018 and 5 October 2020 at the newborn nursery or the outpatient pediatrics clinics affiliated with our academic medical center. This study was approved by the Penn State College of Medicine Institutional Review Board (STUDY00008657). Participating families provided written, informed consent. All participating infants were enrolled within seven days of birth. There were 2487 infants screened for eligibility, 359 who met the criteria, 221 who consented to participate, and 121 who completed the study. Study completion was defined as the contribution of a saliva sample at 6 months and completion of neurodevelopmental screening by a general pediatrician at 18 months (using the Survey of Wellbeing in Young Children; SWYC) and clinical judgement [28]. The primary medical outcome was neurodevelopmental status at 18 months, defined by clinical evaluation by a board-certified clinician. In accordance with SWYC scoring guidelines, infants were dichotomized into two groups, namely “appears to meet age expectations” and “needs review”, using an automated scoring tool within the electronic medical record. General pediatrician clinical assessment was used to confirm “at-risk for neurodevelopmental delay” in those requiring review (*n* = 19), and to identify children “at-risk for neurodevelopmental delay” who appeared to meet age expectations on the SWYC (*n* = 1). Determination of neurodevelopmental status was extracted from the electronic medical record by research staff (Figure 4).

### 4.3. Data Collection

Electronic surveys administered by research staff at the time of enrollment were used to obtain medical/demographic characteristics for all infants. Survey responses were confirmed through review of the electronic medical record. The SWYC was used as the primary screening tool for neurodevelopmental status at 9- and 18-months based on current clinical guidelines recommending the administration of standardized developmental screening tools at these ages [4,29]. Compared with other standardized screening tools for neurodevelopmental status, the SWYC has been shown to display comparable accuracy [28]. The following medical and demographic characteristics were collected: infant biological sex, infant race, infant ethnicity, gestational age (weeks), and birth weight (g). The National Survey of Lead and Allergens in Housing (NSLAH) was used to assess the following social determinants of health that could impact neurodevelopmental outcomes: maternal education, maternal marital status, infant health insurance status, home age, household income, and number of persons living in the household [52].

### 4.4. Sample Collection and Processing

Saliva samples were collected from each infant at six months of age in a non-fasting state. The six-month timepoint was selected to capture a period of rapid motor development within the oropharynx (e.g., babbling, blowing raspberries), while avoiding confounding effects of solid food introduction and teething. This time-point also coincides with regularly scheduled well child visits. Saliva was collected from the sub-lingual and parotid regions by inserting a highly absorbent swab into the oropharynx for 10–20 s. Swabs were immediately placed in nucleic acid stabilizing solution (DNA Genotek, Ottawa, ON, Canada) for downstream RNA analysis. Samples were aliquoted and stored at −80 °C within two weeks of sample collection, per manufacturer instructions. As we have previously reported, RNA was extracted from each sample using the miRNeasy Kit (Qiagen, Inc., Germantown, MD, USA), and RNA quality was assessed with an Agilent Bioanalyzer 2100 (Agilent, Santa Clara, CA, USA) [53,54,55]. RNA sequencing was completed at the SUNY Molecular Analysis Core using the Illumina TruSeq Small RNA Prep protocol and a NextSeq500 instrument (Illumina; San Diego, CA, USA) at a targeted depth of ten million, 50-base, paired-end reads per sample. Reads were aligned to the hg38 build of the human genome using Partek Flow (Partek; St. Louis, MO, USA) and the Bowtie2 aligner. Quantification of mature microRNAs was performed using miRBase 22 annotation. Quality of RNA sequencing results was verified through read quality score and total read count. The miRNA features with consistent detection (raw read counts ≥ 10 in ≥100% of samples) were sum normalized and mean-center scaled prior to statistical analysis. Analysis focused on 4 salivary miRNAs previously found to identify older children (2–6 years) with autism (miR-125a-5p, miR-148a-5p, miR-28-3p, and miR-151a-3p) [27].

### 4.5. Statistical Analysis

Medical and demographic characteristics were compared between infants at risk for neurodevelopmental delay and their peers using a Student’s t-test or a chi square test, as appropriate. A Wilcoxon Rank Sum Test was used to compare saliva miRNAs between groups. False discovery rate correction was used to adjust *p*-values for multiple testing, with adjusted *p* < 0.05 considered to be statistically significant. The miRNAs that displayed differences between groups were assessed for associations with neurodevelopmental score on the SWYC using Spearman’s Rank Correlation testing. Logistic regression was used to assess the ability of saliva miRNA candidates to aid identification infants at risk for neurodevelopmental delay, relative to the clinical standard of care—standardized neurodevelopmental screening with the SWYC at 9 months of age. An initial model employing the 9-month SWYC score was compared against a second model adding salivary levels of miR-151a-3p at 6 months. The ability of each model to account for between-groups variance (measured by McFadden’s R-squared) was reported, and significant gains across additive models (*p* < 0.05) were determined. Discriminative accuracy of each model was visualized on a receiver operator characteristic curve. Cross-validated AUC, sensitivity, and specificity were reported. Interactions between SDOH and miRNA candidates were explored. Finally, the change in salivary miRNA levels between one and six months of age was assessed for a subset of infants (*n* = 47) for whom dual samples were available using a within subjects, non-parametric ANOVA (Friedman’s Test). The purpose of this analysis was to determine if saliva miRNA candidates provided static reflections of neurodevelopment potential (i.e., state biomarker), or dynamic reflections of neurodevelopment that could respond to therapy or environmental factors (i.e., trait biomarker).

## Figures and Tables

**Table 1:** Participant characteristics.

Participant Characteristics, *n* (%)	All (*N* = 121)	At Risk (*n* = 20)	Typical Development (*n* = 101)
*Medical/demographic traits*			
Female sex	71 (59)	11 (55)	60 (59)
Non-white race	30 (25)	6 (30)	24 (24)
Hispanic ethnicity	19 (16)	5 (25)	14 (14)
Gestational age (wks), mean (SD)	39.0 (1)	39.1 (1)	39.0 (1)
Birth weight (g) mean (SD)	3370 (425)	3330 (443)	3378 (424)
*Social determinants of health*			
Maternal high school education ^1^	13 (11)	2 (10)	11 (11)
Single parent household	21 (17)	3 (15)	18 (18)
Public health insurance	17 (14)	1 (5)	16 (16)
Home built prior to 1977	28 (24)	1 (5)	27 (27)
Household income < $25,000	5 (5)	2 (11)	3 (3)
Household size, mean (range)	4 (2–9)	4 (3–5)	4 (2–9)

^1^ Includes some high school, high school diploma, or incomplete college degree. Abbreviations: weeks (wks).

## Data Availability

The RNA sequencing data generated as part of this study are deposited in the Gene Expression Omnibus Repository, accession number GSE192543. GEO repository link: https://www.ncbi.nlm.nih.gov/geo/query/acc.cgi?acc=GSE192543.
